# Roles of miR-17-92 Cluster in Cardiovascular Development and Common Diseases

**DOI:** 10.1155/2017/9102909

**Published:** 2017-01-10

**Authors:** Huanyu Gu, Zhuyuan Liu, Lei Zhou

**Affiliations:** Department of Cardiology, The First Affiliated Hospital, Nanjing Medical University, Nanjing 210029, China

## Abstract

MicroRNAs (miRNAs and miRs) are a large class of noncoding, single-stranded, small RNA molecules. The precise control of their expression is essential for keeping tissue homeostasis and normal development of organisms. Thus, unbalanced expression of miRNAs is a hallmark of many diseases. Two to dozens of miRNAs can form into a miRNA cluster, and the miR-17-92 cluster is one of them. Although firstly described as an oncogenic miRNA cluster, the miR-17-92 cluster has also been found to play critical role in normal cardiac development and cardiovascular disease. This review focuses on the characteristics and functions of miR-17-92 cluster in heart.

## 1. Introduction

MicroRNAs (miRNAs and miRs) are endogenous single-stranded fragments composed by 18–24 nucleotides. A single microRNA can target multiple downstream mRNAs for degradation or translational repression by binding to 3′-untranslated regions (UTRs) and thus mediates the posttranscriptional gene regulation. More than 2,000 miRNAs have been identified up to now; however, the biological functions of many of them are still not sufficiently investigated. Recent studies have revealed that miRNAs participate not only in normal cellular physiology such as differentiation [1] and proliferation [2] but also in the progression of a variety of diseases in heart, lung, immune system, and so forth. For example, miR-1 [3] and miR-27b [4] have been reported to play important role in cardiac cell proliferation, morphogenesis, and conduction. Both miR-126 [5] and miR-143/145 [6] are key regulators of cardiovascular development and differentiation.

miRNAs within a cluster are frequently paralogous with each other in high sequence homology, making them target the unique and common mRNAs, acting as a family. Some clusters have been found to be vital to normal development of organism and pathology of disease. For example, miR-199a-214 cluster takes part in heart failure by facilitating glucose metabolism in failing heart from fatty acid utilization in healthy heart [7].

**Figure 1:**
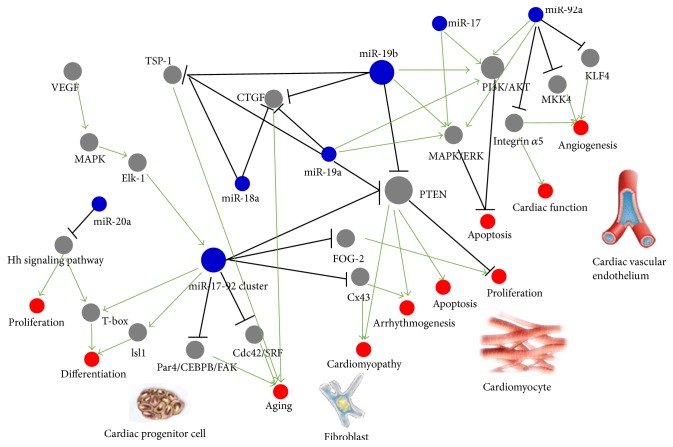
Multiple functions of miR-17-92 cluster in heart.

Among all miRNA clusters, miR-17-92, which was called oncomir-1 at first, is a highly conserved cluster that is essential in adipocyte differentiation [8], lung development [9], angiogenesis [10], tumorigenesis [11], and especially heart development [12, 13]. miR-17-92 cluster has been confirmed to be involved in normal cardiac development and its change took part in the genesis of many cardiovascular diseases, including heart failure [14], cardiomyopathy, arrhythmias, and cardiac hypertrophy [15], while its mechanisms of action in these contexts are not fully understood (Figure 1).

Cardiovascular disease (CVD) is the main threat to human health globally [16], and investigators around the world have spent much effort in the study of this field. Though many great progresses have been made, innovative therapeutics are still needed for cardiovascular diseases. These recent findings in this review about the role of miR-17-92 cluster in heart may shed new light on our understanding and provide potential novel therapeutic targets in the treatment of cardiovascular diseases. Since the cardiomyocytes of most mammalian hearts will not proliferate after birth, the damaged condition is not reversible. MiR-17-92 cluster has important role in proliferation and differentiation of cardiomyocyte. Detecting function of miR-17-92 cluster which had been found to have the capacity of promoting cardiac regeneration would be fundamental for exploring new effective therapies against CVD.

## 2. Biological Role of miR-17-92 Cluster

Pri-miR-17-92 is processed into seven individual mature miRNAs including miR-17-5p, miR-17-3p, miR-18a, miR-19a, miR-19b, miR-20a, and miR-92a, all cleaved by the RNase III enzyme. miR-17-92 cluster is located in the open reading frame 25 (C13orf25) which is an 800-base pair region on chromosome 13 in human genome and on chromosome 14 in mouse genome.

Members of miR-17-92 cluster are expressed in various tissues, while their expression levels depend on different cellular contexts. miR-17-92 cluster is absolutely essential to normal mouse development, as knock-out of miR-17-92 in newborn mice resulted in death shortly after their birth [17]. Overexpression of miR-17-92 would cause mice dying soon after birth with abnormal lung consisting of obvious epithelia hyperplasia and very few normal alveoli [18].

The relationship between miR-17-92 and carcinogenesis is most widely explored. Increased miR-17-92 has been observed in different types of cancer, including lymphoma, lung, colon, and breast [19, 20]. Except for miR-18, other members of miR-17-92 cluster downregulated expression of their downstream target, namely, phosphatase and tensing (PTEN) homolog, which is a well-known tumor suppressor [21]. miR-17-92 cluster has been proven to play critical role in cell growth and differentiation in various cellular contexts by regulating transforming growth factor-*β* (TGF-*β*)/SMAD signaling pathway [22]. For example, high level of miR-17-92 cluster was investigated to have the ability of increasing the number of leukemia stem cells by enhancing cell proliferation and inhibiting cell differentiation, while low level of miR-17-92 cluster had the opposite effect [23]. Recent studies have discovered that miR-17-92 cluster was vital in cardiovascular system, and it could induce cardiomyocytes proliferation not only in embryonic and adult hearts, but also in response to injury by repressing PTEN [13].

## 3. The Role of miR-17-92 Cluster in Cardiovascular Diseases

In recent years, more and more studies have highlighted the role of miR-17-92 in both normal and pathological functions of the heart. Dysregulated expression of miR-17-92 during the cardiovascular morphogenesis led to a lethal arrhythmogenesis and cardiomyopathy, possibly partly through repressing PTEN and gap junction gene Cx43 [15].

### 3.1. miR-17-92 Cluster and Congenital Heart Defect (CHD)

CHD is a common clinical disease of newborns with different phenotypes, and it accounts for about 25% of neonatal mortality within one month after birth and nearly 40% of perinatal mortality [24]. The main cause of CHD is impaired cardiac development such as abnormal differentiation of cardiac progenitor cell (CPC) into cardiomyocyte and unusual cardiac proliferation, and miR-17-92 has been found to take part in these processes.

### 3.2. Differentiation

Loss-of-function of miR-17-92 resulted in aberrant differentiation from CPCs to normal cardiomyocytes via repressing the function of cardiac progenitor gene Isl1 in the process of embryonic cardiac development in mice. Except for differentiation, miR-17-92 also influenced cardiac morphogenesis by repressing T-box genes [25]. miR-20a overexpression in P19 cells has been found to be able to inhibit differentiation and proliferation; meanwhile it accelerated apoptosis through Hh signaling pathway which has been found to induce cardiac differentiation during the cardiac embryonic development [26].

### 3.3. Proliferation

miR-17-92 is absolutely essential in cardiac development, both in embryonic and postnatal hearts, and the previous study has reported that cardiogenesis was disturbed in mice lacking miR-17-92 [17]. Proliferation of endothelial cells of cardiac blood vessels could be supported by high level of miR-17-92 cluster, which was triggered by vascular endothelial growth factor (VEGF) through mitogen activated protein kinase (MAPK) activation and Elk-1 phosphorylation signaling pathway. Moreover, loss of endothelial miR-17-92 cluster led to vascular impairment [27]. Another study based on embryonic cardiomyocytes reported that overexpression of miR-17-92 would reduce the cell proliferation through posttranscriptional repression of Friend of Gata-2 (FOG-2, a nuclear corepressor protein), which is a critical factor for cardiac development. Moreover, FOG-2 could partially rescue the abnormal proliferation induced by miR-17-92 [12]. In addition, miR-17-92 cluster member miR-19 promoted cardiomyocyte proliferation through direct downregulation of PTEN in vitro [21]. In nonmammal zebrafish, an increasing overexpression of miR-19b in embryos induced more abnormal cardiac development which is characterized by defected cardiac looping, slower heart rate, and edematous pericardium. Moreover, overexpression of miR-19b induced the inhibition of Wnt activity by targeting ctnnb1. Wnt is a key regulator of cardiac progenitor cell differentiation and self-renewal, and ctnnb1 is important to asymmetry of heart [28].

### 3.4. miR-17-92 Cluster and Coronary Heart Disease

Coronary heart disease is the third cause of death in developing countries and ranks the first in developed countries. Novel therapies for coronary heart disease are highly needed.

### 3.5. Myocardial Infarction (MI)

The relationship between miR-17-92 and MI has been studied a lot in the past few years. miR-17-92 cluster regulates different processes during cardiac repair, and its members may be regarded as worthy therapeutic targets in the treatment of ischemic heart diseases.

miR-17-92, especially the key component miR-19, could promote the proliferation of cardiomyocytes to help protect heart from the ischemic injury which was caused by MI through the major target PTEN [13]. Using gain and loss-of-function experiments, miR-92a was found to be able to restrain angiogenesis and migration of endothelial cells in adult mice heart, while inhibition of miR-92a enhanced angiogenesis and cardiac function after carotid arteries injury and MI by regulating expression level of tumor suppressor MKK4 and KLF4 in endothelial cells [29]. After induction of MI and limb ischemia, systemic infusion of antagomirs targeting miR-92a led to enhanced cardiac function and blood vessels growth marked by capillary density of ischemic tissues by targeting proangiogenic factors integrin subunit alpha5 [30]. In a mouse model of MI, gain-of-function of miR-17-92 in heart led to upregulated proliferation of cardiomyocytes and improved cardiac function. Moreover, scar size was reduced apparently and the numbers of proliferating cardiomyocytes at the border zone were increased compared with controls. Besides that, loss-of-function of miR-17-92 had the inverse effects [21].

### 3.6. Myocardial Ischemia/Reperfusion (I/R) Injury

Myocardial ischemia/reperfusion is a model imitating cardiac surgery and injury of myocardial infarction, and its major mechanism is perceived as apoptosis [31]. I/R injury can induce postprocedural cardiac dysfunction, arrhythmias, and subsequent operative mortality. miR-15 [32], miR-210 [33], and miR-494 [34] were found to take part in the cardiac I/R injury. In the hypoxia-induced apoptotic tumor tissues, miR-17-92 cluster was documented to have the antiapoptotic ability [35].

miR-17-92 cluster can protect the heart by negatively controlling the apoptosis and alleviating I/R injury. miR-17, miR-19, and miR-92 played role in resistance to apoptosis by directly inhibiting proapoptotic protein through the MAPK/ERK and PI3 K/AKT signaling pathways which are important in regulating cell survival [36]. A recent study has found that, in a mouse model of cardiac I/R injury, miR-19b was the only member of the miR-17-92 cluster downregulated in the infarct zone. Moreover, overexpression of miR-19b could improve the survival of rat H9C2 cardiomyocytes and alleviate the apoptosis induced by H_2_O_2_ [37].

### 3.7. miR-17-92 Cluster and Cardiac Aging

Cardiac aging is a common cause of many cardiovascular diseases. miR-18a, miR-19a, and miR-19b were three of the most decreased miRNAs in hearts of old mice and aged cardiomyocytes, and their targets were downregulated. One target was thrombospondin-1 (TSP-1), and the other was extracellular matrix proteins connective tissue growth factor (CTGF) [14]. Based on miRNA arrays of mice heart tissues, members of miR-17-92 cluster apparently targeted multiple components of Cdc42-SRF signaling pathway which is classically activated in aging process [38]. Transgenic expression of miR-17 in mouse has been reported to prevent cardiac fibroblast senescence and cardiac senescence marked by lower intensities of *β*-gal staining by directly targeting Par4, thus activating the Par4-CEBPB-FAK senescence signaling pathway, which takes important part in controlling cell apoptosis, growth, survival, and epithelial-to-mesenchymal transition [39].

## 4. Conclusions and Perspectives

It is becoming increasingly obvious that miRNAs and miRNA clusters are necessary in maintaining the organism's normal function, the response to environmental stimuli, the development under pathological conditions, and the process of aging. In this review, we summarized the current knowledge about miR-17–92 cluster and its roles in heart, including aspects of physiology and pathology (Table 1).

As the patients of CVD are becoming younger in average age, new medications are needed urgently. Better understanding of the roles of miR-17-92 cluster in heart will truly help us open up a new prospect for treatment of CVD. Since the expression levels of members of miR-17-92 are different from normal individuals, for example, miR-19b was downregulated in the I/R injured mouse models and miR-18a, miR-19a, and miR-19b were decreased in old mice and aged cardiomyocytes, the miR-17-92 may become potential diagnostic markers of cardiac disease. Previous studies revealed the antitumorigenic effects of miR-17-92 in tumor cells. Usage of intravenous delivery of anti-miR-17-92 resulted in blockage of medulloblastoma tumor growth in immune-compromised mice [40]. Research in vivo animal models have shed light on the potentiality of targeting miR-17-92 components therapeutically. As for the cardiac system, a new exciting finding demonstrated that resident cardiomyocytes can be induced to undergo cytokinesis after reentering the cell cycle, while the underlying molecular mechanisms are still unknown. The studies summarized in this review indicate that miR-17-92-based therapeutics can be used to induce cardiomyocytes to reenter cell cycle to recover cardiac injury such as myocardial infarction which can induce heart failure. However, in order to be sure of the safe therapeutic application of miR-17-92 for the treatment of cardiac diseases, side effects of miR-17-92 mimics and antagonists in animal models still need to be addressed by further works.

## Figures and Tables

**Table 1:** The function of miR-17-92 cluster and the relationship with cardiovascular disease.

Cardiac parameter	Effects of miR-17-92	Target	Reference
Cardiomyocyte differentiation	Interference cardiac morphogenesis Promote differentiation	T-box Isl1	[25]

Cardiomyocyte proliferation	MiR-19 promotes cardiomyocyte growth and proliferation	Wnt signaling pathway	[21]
	Support proliferation of endothelial cells of cardiac blood vessels	MAPK, Elk-1	[27]

MI	Upregulate proliferation of cardiomyocytes, improve cardiac function, and reduce scar size		[21]
	MiR-19 protects ischemic injury	PTEN	[13]
	MiR-92a restrains endothelial cell angiogenesis	MKK4, KLF4	[29]

IRI	Resistance to apoptosis	MAPK/ERK, PI3K/AKT signaling pathway	[36]
	MiR-19b alleviate apoptosis and improve survival of H9C2 cardiomyocyte	PTEN	[37]

Aging	Inhibit aging process	Cdc42-SRF signaling pathway	[38]
	Prevent cardiac fibroblast senescence	Par4-CEBPB-FAK signaling pathway	[39]
